# Risk Factors and Outcomes of Acute Myocardial Infarction in a Cohort of Antiphospholipid Syndrome

**DOI:** 10.3389/fcvm.2022.871011

**Published:** 2022-07-05

**Authors:** Yuzhou Gan, Yawei Zhao, Gongming Li, Hua Ye, Yunshan Zhou, Chang Hou, Lan Wang, Jianping Guo, Chun Li

**Affiliations:** ^1^Beijing Key Laboratory for Rheumatism and Immune Diagnosis (BZ0135), Department of Rheumatology and Immunology, Peking University People's Hospital, Beijing, China; ^2^Center of Clinical Immunology, Peking University, Beijing, China; ^3^Department of Rheumatology and Immunology, Luohe Central Hospital, Luohe, China; ^4^Department of Rheumatology, Linyi Traditional Chinese Medicine Hospital, Linyi, China; ^5^Beijing Key Laboratory of Early Prediction and Intervention of Acute Myocardial Infarction, Department of Cardiology, Peking University People's Hospital, Beijing, China

**Keywords:** acute myocardial infarction, antiphospholipid syndrome, risk factor, thrombosis, atherosclerosis

## Abstract

**Background:**

Antiphospholipid syndrome (APS) is a disorder associated with thromboembolic diseases, including acute myocardial infarction (AMI). Given that AMI is a relatively common condition with poor prognostic features, identification of risk factors for AMI in APS is important.

**Methods:**

A retrospective cohort study was performed consisting of 332 patients with APS, and 239 patients with thrombotic APS were finally included. Patients were followed up in the outpatient department for 5 years. Clinical data and laboratory parameters were analyzed to identify the risk factors for AMI in APS. The primary and secondary clinical outcomes were all-cause mortality and recurrence of thrombosis, respectively.

**Results:**

AMI was observed in 12.1% (29/239) of patients with APS. Compared to patients without AMI, patients with AMI had multiple organ thrombosis (55.1 vs. 34.3%, *p* = 0.029), recurrent thrombosis (58.6 vs. 34.3%, *p* = 0.011), a higher incidence of atherosclerosis (62.1 vs. 23.8%, *p* < 0.001), higher neutrophil count (×10^9^/L) [4.68 (3.25, 8.17) vs. 3.71 (2.64, 5.80), *p* = 0.036], longer QT interval (ms) [438 ms (423, 454) vs. 425 ms (410, 446), *p* = 0.016], and fewer venous thrombosis events (27.6 vs. 63.3%, *p* < 0.001). Multivariate logistic regression analysis (adjusted for age and gender) identified several factors that were positively associated with AMI, including multiple organ thrombosis [odds ratio (OR) 8.862, 95% confidence interval (CI): 1.817–43.212, *p* = 0.007), atherosclerosis (OR 5.397, 95%CI: 1.943–14.994, *p* = 0.001), and elevated neutrophil count (>6.3 ×10^9^/L) (OR 3.271, 95%CI: 1.268–8.440, *p* = 0.014). The venous thrombosis was negatively associated with AMI (OR 0.106, 95%CI: 0.036–0.314, *p* < 0.001). Kaplan–Meier analysis revealed that the recurrence rates of arterial thrombosis differed significantly between patients with AMI and those without AMI [hazard ratio (HR) = 3.307, *p* = 0.038].

**Conclusion:**

Atherosclerosis, multiple organ thrombosis, an increased number of neutrophils are variables positively associated with AMI in APS, and venous thrombosis had a negative association with AMI. AMI only predicts the subsequent recurrence of arterial thrombosis. These findings suggest that distinct pathophysiological mechanisms may exist and contribute to the development of venous or arterial thrombotic APS.

## Introduction

Antiphospholipid syndrome (APS) is a prothrombotic autoimmune disease characterized by recurrent thrombosis and/or obstetric events in the presence of antiphospholipid antibodies. Coronary artery disease is one of the main cardiac manifestations of APS, and 2.8–5.5% of patients with acute myocardial infarction (AMI) are young individuals with AMI secondary to APS (1). In one study of patients with APS with a low pre-test probability for cardiovascular events, the prevalence of myocardial scarring detected by cardiac MRI was surprisingly high (11%) (2). Furthermore, Cervera et al. reported that 10% of patients with primary APS died of AMI (3). In addition, poor outcomes of AMI in autoimmune diseases have been confirmed in numerous studies (4, 5). Thus, AMI is a relatively common condition with a poor prognosis in APS. However, to date, there has been no cohort study focusing on the clinical and laboratory features and prognosis of APS patients with AMI, including subsequent death and recurrent thrombosis. Although the control of traditional cardiovascular disease (CVD) risk factors in APS has been emphasized by recommendations as overarching principles (6), it remains unclear whether CVD risk factors fully contribute to AMI in APS. In addition, the accelerated atherosclerosis and increased prevalence of CVD cannot be completely explained by traditional risk factors or the use of glucocorticoids in other autoimmune diseases (7). The aim of the present study was to characterize and identify the risk factors for AMI in APS.

## Materials and Methods

### Patients

This was a single-center retrospective cohort study performed at the Department of Rheumatology, Peking University People's Hospital. A total of 332 patients with APS were consecutively enrolled between July 2009 and January 2021. The diagnosis of APS was confirmed by two rheumatologists (YZG and YWZ) according to the 2006 Sapporo criteria (8) or catastrophic antiphospholipid syndrome (CAPS) according to the current diagnostic criteria (9). Another inclusion criterion was the disease onset age, which was >18 years. The study was approved by the ethics committee of Peking University People's Hospital (2019PHB253-01) and complied with the Declaration of Helsinki guidelines.

### Clinical and Laboratory Data Collection

Baseline demographics and clinical and laboratory characteristics were obtained from the electronic medical records at the time of APS diagnosis. Venous thromboembolic events (e.g., deep venous thrombosis of the upper limbs of the legs, visceral venous thrombosis, and/or pulmonary embolism) were confirmed by limb ultrasound, pulmonary computed tomography (CT) or scintigraphy (ventilation/perfusion), abdominal pelvic CT scan, and vessel angiography as indicated. Arterial thrombotic events (e.g., peripheral arterial thrombosis, acute cerebral infarction, and/or visceral arterial thrombosis) were diagnosed using typical clinical pictures with positive arteriography [e.g., leg or upper limb ultrasound, CT, or magnetic resonance angiography (MRA)] and surgery. Multiple organ thrombosis was defined as the involvement of at least two organ systems during the course of the disease. The adjusted global antiphospholipid syndrome score (aGAPSS) was calculated for each patient by adding the points corresponding to the risk factors, excluding antibodies to phosphatidylserine/prothrombin (aPS/PT) that are not routinely tested in most clinical laboratories, as previously described (10). The aGAPSS ranged from 0 to 17.

### Arterial and Cardiovascular Assessment

Atherosclerosis was defined as the presence of any plaque in the carotid or femoral arteries, which were tested using B-mode and Doppler ultrasound examination with an Aplio 500 system (Canon Medical Systems Corp., Tochigi, Japan) with a 14L5 transducer or a Logic E9 system (GE Healthcare, Chicago, IL, USA) with a 9L transducer. Electrocardiography (ECG) was performed using a MAC 5500 HD resting ECG system (GE Healthcare, Wauwatosa, USA). Transthoracic echocardiogram (TTE) evaluation was performed using either an iE33 (S5-1 probe, Philips Medical Systems, Andover, Massachusetts, USA) or a Vivid E9 (GE Vingmed, Horten, Norway, UK) scanner with a 2.5–3.5 MHz transducer. AMI was diagnosed according to the fourth universal definition of myocardial infarction and classified as ST segment elevation myocardial infarction (STEMI), non-ST segment elevation myocardial infarction (NSTEMI), and unstable angina (UA) (11). Myocardial infarction with non-obstructive coronary arteries (MINOCA) was defined as no coronary artery stenosis > 50% in any potential infarct-related artery without other clinically overt specific causes for the acute presentation (12).

### Follow-Up Procedure and Clinical Outcomes

Patients were followed up for 5 years or monitored up to 31 December 2021 if the patients were enrolled after 30 December 2016 in outpatient services. Follow-up information was also obtained from electronic medical records or regular medical examination reports. Medication data were recorded, including sustained anticoagulation treatment, antiplatelet therapies, corticosteroids, immunosuppressants, and statins. If patients received warfarin, the international normalized ratio (INR) was documented every 3 months and the mean INR was calculated. The primary clinical outcome was all-cause mortality (defined as the time from recruitment to death from any cause). The second clinical outcome was the recurrence of thrombosis. Thrombotic events were independently adjudicated by two investigators (YZG and YWZ).

### Statistical Analysis

Continuous data with normal distribution were expressed as the mean ± standard deviation, and differences between groups were analyzed using one-way ANOVA. Continuous data with skewed distribution were expressed as medians (P25, P75), and differences between groups were analyzed using the Kruskal–Wallis test. Dichotomous variables were reported as frequencies (percentages), and differences between groups were compared using the chi-square test (or Fisher exact test when appropriate). Univariate and multivariate logistic regression analyses with an imputed dataset were adopted to identify risk factors for AMI, adjusted for age and sex. The variables assessed in the group differences were entered as independent variables in the univariate logistic regression analysis when the *p* < 0.1. The variables assessed in the univariate regression analysis were entered as independent variables in the multivariate logistic regression analysis when the *p* < 0.1. Survival and recurrence of thrombosis were estimated using the Kaplan–Meier method, and differences were evaluated using a stratified log-rank test. Data analyses were performed using SPSS 23.0 for Windows. Two-sided *p* < 0.05 was considered statistically significant.

## Results

### Study Population

**Figure 1:**
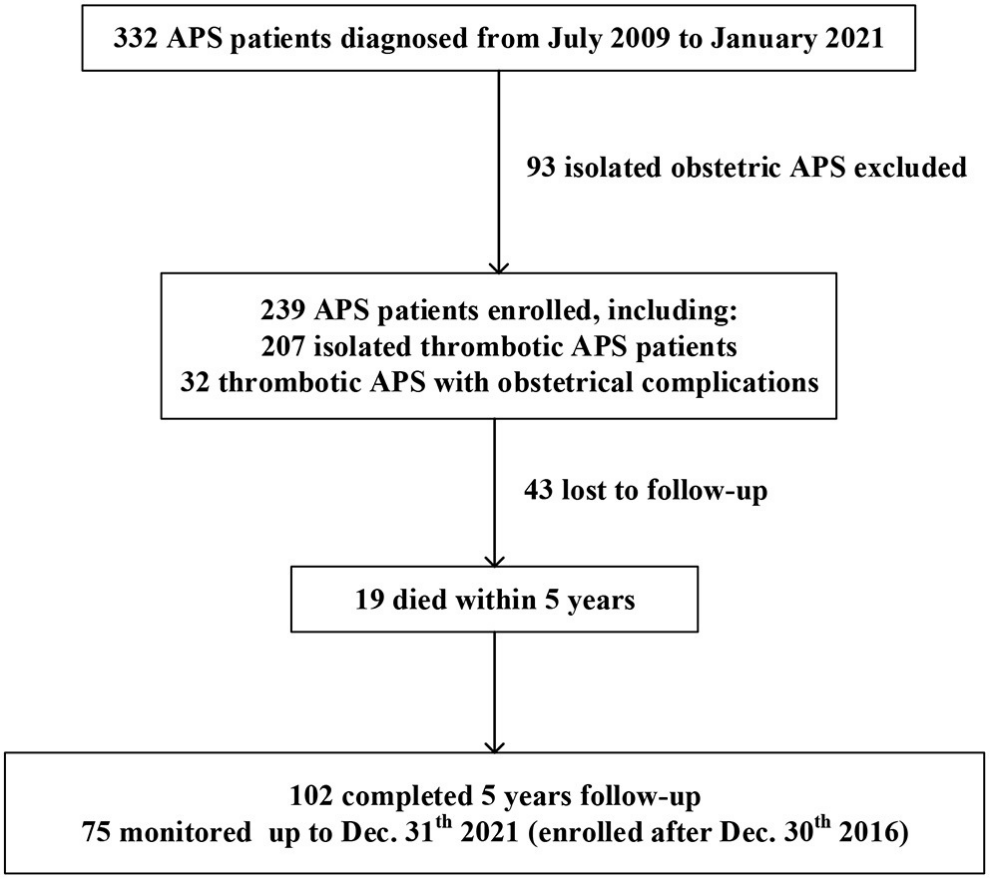
A flow chart of our retrospective study design. Of the 332 APS patients recruited, 239 thrombotic APS patients were enrolled.

Of the 332 APS patients, 93 had isolated obstetric APS, 207 had isolated thrombotic APS, and 32 had thrombotic APS with obstetric complications. A flow diagram of the individuals at each stage is shown in Figure 1. A total of 239 patients with thrombotic APS (207 isolated thrombotic and 32 thrombotic APS with obstetrical complications) were enrolled in our cohort. Follow-up data were available for 196 patients (82.0%) with an overall median follow-up time of 4.5 years. Of the 196 patients, 102 (52.0%) completed a 5-year follow-up and 21 (10.7%) died within 5 years.

### Clinical Profiles of APS Patients With AMI

The incidence of AMI was 12.1% (29/239) and detailed clinical profiles are shown in Table 1. Nine patients with AMI were male, and the average age of AMI onset was 44.6 years (Table 2). Of the 29 patients with AMI, 14 (48.3%) had STEMI, 5 (17.2%) had NSTEMI, and 10 (34.5%) had UA. In total, 13 patients with APS (44.8%) developed AMI before APS confirmation (range, 7 months to 30 years), 9 patients (31.0%) developed AMI after APS diagnosis (range, 1 month to 5 years), and 7 patients (24.2%) presented with AMI simultaneously with APS (confirmations were performed after 12 weeks). In total, 1 patient (3.4%) underwent coronary artery bypass grafting, 1 patient (3.4%) underwent thrombolysis, 9 patients (31.0%) underwent percutaneous coronary intervention, and the remaining 18 patients (62.2%) received conservative medical treatment. Of the 22 patients who underwent coronary angiography, 7 (31.8%) were MINOCA, including UA, STEMI, and NSTEMI (5, 2, and 1, respectively). One of the patients with AMI died within the first 30 days.

**Table 1:** Detailed clinical profiles of APS patients with AMI.

**No**.	**Onset age/sex**	**AMI subtype**	**MINOCA**	**Time from AMI onset to APS confirmation**	**Thrombotic events previous to APS**	**Treatment strategies**	**Outcome during the first 30 days**
1	52/F	UA	No	12 months before	None	Emergent PCI	Survival
2	39/F	UA	Yes	Simultaneously	None	Conservative treatment	Survival
3	34/M	STEMI	No	12 months after	None	Emergent PCI	Survival
4	44/M	STEMI	No	7 years after	Acute cerebral infarction	Emergent PCI	Survival
5	49/M	STEMI	No	30 years before	Acute cerebral infarction	Conservative treatment	Survival
6	68/M	NSTEMI	No	12 months before	Acute cerebral infarction	Conservative treatment	Survival
7	40/M	UA	/	9 years before	None	Conservative treatment	Survival
8	41/F	UA	Yes	4 years before	None	Conservative treatment	Survival
9	25/F	STEMI	Yes	Simultaneously	None	Conservative treatment	Survival
10	74/F	UA	/	12 months after	None	Conservative treatment	Survival
11	63/F	NSTEMI	Yes	1 months after	None	Conservative treatment	Survival
12	43/F	UA	No	6 months after	Acute cerebral infarction	Emergent PCI	Survival
13	47/F	STEMI	No	1 months after	Iliac artery thrombosis	Emergent PCI	Survival
14	53/F	STEMI	No	Simultaneously	None	CABG	Survival
15	39/F	STEMI	/	22 years before	Acute lacunar infarction	Conservative treatment	Survival
16	85/M	STEMI	No	4 years before	None	Conservative treatment	Survival
17	20/M	STEMI	No	12 months after	Deep vein thrombosis and pulmonary embolism	Emergent PCI	Survival
18	78/F	NSTEMI	/	5 years before	Aortic thrombosis, deep vein thrombosis and pulmonary embolism	Conservative treatment	Survival
19	60/F	UA	Yes	7 months before	Acute cerebral infarction and deep vein thrombosis	Conservative treatment	Survival
20	24/F	UA	/	Simultaneously	Deep vein thrombosis and pulmonary embolism	Conservative treatment	Died
21	36/M	UA	Yes	5 years before	Deep vein thrombosis and pulmonary embolism	Conservative treatment	Survival
22	71/F	STEMI	No	Simultaneously	Acute lacunar infarction	Conservative treatment	Survival
23	26/F	STEMI	Yes	6 months after	None	Thrombolysis	Survival
24	20/F	STEMI	No	Simultaneously	None	Conservative treatment	Survival
25	48/F	STEMI	No	Simultaneously	None	Emergent PCI	Survival
26	29/M	STEMI	No	8 years before	Deep vein thrombosis	Emergent PCI	Survival
27	67/F	NSTEMI	/	4 years before	Deep vein thrombosis	Conservative treatment	Survival
28	73/F	UA	/	6 years before	Deep vein thrombosis and pulmonary embolism	Conservative treatment	Survival
29	63/F	NSTEMI	No	4 years after	Acute cerebral infarction	Emergent PCI	Survival

*APS, antiphospholipid syndrome; AMI, acute myocardial infarction; MINOCA, myocardial infarction with non-obstructive coronary arteries; STEMI, ST segment elevation myocardial infarction; NSTEMI, non-ST segment elevation myocardial infarction; UA, unstable angina; PCI, percutaneous coronary intervention; CABG, coronary artery bypass graft*.

**Table 2:** Baseline characteristics of APS patients with or without AMI.

	**Overall (*n* = 239)**	**With AMI (*n* = 29)**	**Without AMI (*n* = 210)**	***p***
Age of onset (years)	45.0 ± 17.4	47.9 ± 17.8	44.6 ± 17.3	0.342
Gender (M/F)	29/210	9/20	66/144	0.966
Clinical criteria manifestation				
Venous thrombosis, *n* (%)	141 (58.9)	8 (27.6)	133 (63.3)	**<0.001**
Pregnancy morbidity, *n* (%)	32 (13.4)	4 (13.8)	28 (13.3)	0.946
Primary APS, *n* (%)	93 (38.9)	13 (44.8)	80 (38.1)	0.486
Multiple organ thrombosis, *n* (%)	88 (36.8)	16 (55.1)	72 (34.3)	**0.029**
Thrombotic events≥2, *n* (%)	89 (37.2)	17 (58.6)	72 (34.3)	**0.011**
Laboratory criteria manifestation				
aCL + (*n*, %)	174 (72.8)	22 (75.9)	152 (72.4)	0.693
aCL (U/L)	19.1 (8.28, 52.96)	31.2 (10.5, 64.8)	17.3 (8.2, 46.9)	0.140
Anti-β2 GPI +, *n* (%)	147 (61.5)	21 (72.4)	126 (60)	0.198
Anti-β2 GPI (RU/mL)	39.47 (11.13, 115.43)	53.9 (17.2, 150.4)	38.6 (9.9, 110.6)	0.183
LA +, *n* (%)	173 (72.4)	23 (79.3)	150 (71.4)	0.374
LA	1.40 (1.22, 1.78)	1.57 (1.33, 1.98)	1.37 (1.22, 1.76)	0.148
High-risk aPL, *n* (%)	177 (74.0)	25 (86.2)	152 (72.4)	0.111
Triple positive, *n* (%)	95 (39.7)	15 (51.7)	80 (38.1)	0.160
Comorbidities				
Smoking, *n* (%)	49 (20.5)	9 (31.0)	40 (19.0)	0.134
Dyslipidemia, *n* (%)	51 (21.3)	9 (31.0)	42 (20)	0.174
Hypertension, *n* (%)	90 (37.6)	12 (41.4)	78 (37.1)	0.659
Atherosclerosis, *n* (%)	68 (28.4)	18 (62.1)	50 (23.8)	**<0.001**
Diabetes, *n* (%)	36 (15.1)	6 (20.7)	30 (14.3)	0.366
Chronic pulmonary disease, *n* (%)	5 (2.1)	2 (6.9)	3 (1.4)	0.054
Chronic kidney disease, *n* (%)	23 (9.6)	1 (3.4)	22 (10.5)	0.229
Hyperuricemia, *n* (%)	29 (12.1)	1 (3.4)	28 (13.3)	0.126
Accompanied autoimmune disease, *n* (%)	144 (60.2)	16 (55.2)	128 (60.1)	0.551
Laboratory manifestations				
White blood cell count (×10^9^/L)	5.93 (4.42, 8.10)	6.94 (5.08, 9.92)	5.65 (4.38, 7.88)	0.054
Neutrophils (×10^9^/L)	3.88 (2.67, 6.10)	4.68 (3.25, 8.17)	3.71 (2.64, 5.80)	**0.036**
Hemoglobin (g/L)	120 (100, 136)	123 (96.5, 143)	120 (101, 134)	0.851
Platelet (×10^9^/L)	148 (72, 218)	150 (70, 221)	147 (75, 210)	0.962
Total cholesterol (mmol/L)	4.20 (3.49, 5.04)	3.95 (3.33, 5.32)	4.24 (3.49, 5.00)	0.779
Triglyceride (mmol/L)	1.43 (1.09, 2.06)	1.47 (1.09, 2.08)	1.42 (1.09, 2.06)	0.624
Low density lipoprotein cholesterol (mmol/L)	2.40 (1.98, 3.14)	2.28 (1.77, 3.08)	2.46 (1.99, 3.15)	0.291
High density lipoprotein cholesterol (mmol/L)	1.08 ± 0.42	1.20 ± 0.54	1.07 ± 0.42	0.097
C reactive protein (mg/L)	3.7 (1.1, 16.4)	3.0 (1.12, 7.0)	4.2 (1.1, 18.8)	0.187
Erythrocyte sedimentation rate (mm/h)	23 (9, 57)	23 (9, 58)	21 (11, 48)	0.956
Immunoglobulin A (g/L)	2.57 (1.60, 3.62)	2.20 (1.61, 3.04)	2.59 (1.60 3.70)	0.503
Immunoglobulin G (g/L)	13.60 (10.30, 17.50)	13.50 (11.25, 17.20)	13.60 (10.07, 17.50)	0.734
Immunoglobulin M (g/L)	1.12 (0.69, 1.74)	0.91 (0.56, 1.47)	1.13 (0.72, 1.77)	0.169
Complement 3 (g/L)	0.79 (0.59, 1.00)	0.74 (0.55, 1.07)	0.80 (0.60, 1.00)	0.545
Complement 4 (g/L)	0.16 (0.11, 0.24)	0.15 (0.08, 0.20)	0.17 (0.11, 0.24)	0.127
ANA≥1:80, n (%)	133 (55.6)	16 (55.2)	117 (55.7)	0.956
aGAPSS	10 (7, 13)	13 (9, 14)	10 (7, 13)	0.214

*APS, antiphospholipid syndrome; aCL, anticardiolipin antibody; Anti-β2GPI, anti-β2-glycoprotein I antibody; LA, lupus anticoagulant; AMI, acute myocardial infarction; GPI, glycoprotein domain I; aPL, antiphospholipid antibody; ANA, antinuclear antibody; aGAPSS, adjusted Global Antiphospholipid Syndrome Score. The bold values means significantly different (p <0.05)*.

### Comparison Between APS Patients With and Without AMI

Next, we compared baseline demographics, clinical characteristics, and laboratory parameters between patients with and without AMI (Table 2). Patients with AMI had multiple organ thrombosis (55.1 vs. 34.3%, *p* = 0.029), recurrent thrombosis (58.6 vs. 34.3%, *p* = 0.011), higher incidence of atherosclerosis (62.1 vs. 23.8%, *p* < 0.001), higher neutrophil count (×10^9^/L) [4.68 (3.25, 8.17) vs. 3.71 (2.64, 5.80), *p* = 0.036], and fewer venous thrombosis events (27.6 vs. 63.3%, *p* < 0.001). Demographic data, other clinical or laboratory features, and aGAPSS scores did not show any significant differences between the two groups. Supplementary Table 1 showed the detailed profiles of accompanied autoimmune diseases.

For cardiac manifestations, the QTc interval (ms) was significantly longer in APS patients with AMI [438 (423, 454) vs. 425 (410, 446), *p* = 0.016]. However, significant differences were not observed in other cardiac features, including arrhythmia, myocardial hypertrophy, echocardiographic parameters, and percentage of hydroxychloroquine usage (shown in Table 3).

**Table 3:** Comparison of cardiac manifestations between APS with and without AMI.

	**Overall**	**With AMI**	**Without AMI**	***p***
Arrhythmia, *n* (%)	24 (10.0)	5 (17.2)	19 (6.1)	0.169
Myocardial hypertrophy, *n* (%)	20 (8.4)	2 (6.9)	18 (5.8)	0.734
**Electrocardiogram**, ***n*** **(%)**	**229 (100)**	**27 (11.8)**	**202 (88.2)**	
QTc (ms)	427 (411, 448)	438 (423, 454)	425 (410, 446)	**0.016**
**Echocardiographic parameters**, ***n*** **(%)**	**210 (100)**	**26 (12.4)**	**184 (87.6)**	
Left Ventricular Ejection Fraction <50%, *n (*%)	5 (2.4)	1 (3.8)	4 (2.2)	0.487
Mitral E/A	1.08 (0.75, 1.4)	0.82 (0.70, 1.35)	1.10 (0.77, 1.40)	0.196
Left atrial diameter (cm)	3.4 (3, 3.8)	3.3 (3, 3.7)	3.4 (3, 3.8)	0.927
IVSDd (cm)	0.88 (0.8, 0.98)	0.9 (0.85, 1.00)	0.87 (0.80, 0.97)	0.162
Left ventricular end diastolic diameter (cm)	4.7 (4.47, 5.1)	4.85 (4.59, 5.1)	4.84 (4.59, 5.1)	0.314
Left ventricular end systolic diameter (cm)	3.0 (2.7, 3.21)	3.08 (2.78, 3.38)	3 (2.7, 3.2)	0.183
Left ventricular posterior wall thickness (cm)	0.87 (0.80, 0.95)	0.86 (0.82, 1.00)	0.87 (0.79, 0.95)	0.320
Maximum transarotic valve flow rate (cm/s)	123.2 (108.3, 143.63)	120.17 (103.35, 145.50)	123.4 (108.65, 143.65)	0.994
Peak pressure gradients across aortic valve (mmHg)	6.1 (4.7, 8.3)	5.8 (4.3, 8.5)	6.1 (4.7, 8.3)	0.912
Systolic pulmonary arterial pressure (mmHg)				0.208
≥45, *n* (%)	10	3	7	
25~44, *n* (%)	46	6	40	
<25, *n* (%)	154	17	137	

*APS, antiphospholipid syndrome; AMI, acute myocardial infarction; IVSDd, intraventricular septum diameter in diastole. The bold values means significantly different (p <0.05)*.

### Risk Factors for AMI in APS

In logistic regression analysis, the variables assessed in the previous analysis were entered as independent variables with a cut-off *p* < 0.1. Results of the univariate and multivariate logistic analyses are shown in Table 4. In univariate logistic analysis, several variables were positively associated with AMI, including multiple organ thrombosis [odds ratio (OR) 2.715, 95% confidence interval (CI): 1.230–5.995, *p* = 0.013], recurrent thrombosis (OR 2.359, 95%CI: 1.076–5.174, *p* = 0.032), atherosclerosis (OR 5.236, 95%CI: 2.319–11.82, *p* < 0.001), and elevated neutrophil count (>6.3 ×10^9^/L) (OR 3.000, 95%CI: 1.328–6.780, *p* = 0.008). Interestingly, venous thrombosis was negatively associated with AMI (OR 0.221, 95%CI: 0.093–0.522, *p* = 0.001). With age and sex adjustment, the multivariate logistic models revealed that multiple organ thrombosis (OR 8.862, 95%CI: 1.817–43.212, *p* = 0.007), atherosclerosis (OR 5.397, 95%CI: 1.943–14.994, *p* = 0.001), and elevated neutrophil count (>6.3 ×10^9^/L) (OR 3.271, 95%CI: 1.268–8.440, *p* = 0.014) might be risk factors for AMI, and venous thrombosis (OR 0.106, 95%CI: 0.036–0314, *p* < 0.001) might be protective factors for AMI. Traditional CVD risk factors (smoking, diabetes mellitus, hypertension, dyslipidemia, chronic kidney disease, and hyperuricemia) were not associated with AMI in APS.

**Table 4:** Risk factors for AMI in APS by logistic models.

**Variables**	**Univariate analysis**			**Multivariate analysis**		
	**B**	**OR (95%CI)**	***p***	**B**	**OR (95%CI)**	***p***
**APS with AMI**						
Age of onset	0.011	1.011 (0.989, 1.034)	0.341	0.007	1.007 (0.978, 1.038)	0.638
Male	0.018	1.019 (0.440, 2.357)	0.966	−0.786	0.456 (0.160, 1.300)	0.142
Venous thrombosis	−1.512	0.221 (0.093, 0.522)	**0.001**	−2.247	0.106 (0.036, 0314)	**<0.001**
Multiple organ thrombosis	0.999	2.715 (1.230, 5.995)	**0.013**	2.182	8.862 (1.817, 43.212)	**0.007**
Thrombotic events≥2	0.858	2.359 (1.076, 5.174)	**0.032**	−0.487	0.614 (0.142, 2.661)	0.515
Atherosclerosis	1.656	5.236 (2.319, 11.824)	**<0.001**	1.686	5.397 (1.943, 14.994)	**0.001**
Chronic pulmonary disease	1.631	5.111 (0.817, 31.977)	0.081			
Smoking	0.648	1.912 (0.810, 4.514)	0.139			
Diabetes mellitus	0.448	1.565 (0.589, 4.162)	0.369			
Hypertension	0.178	1.195 (0.542, 2.633)	0.659			
Dyslipidemia	0.588	1.800 (0.765, 4.238)	0.179			
Chronic kidney disease	−1.187	0.305 (0.040, 2.354)	0.255			
Hyperuricemia	−1.460	0.232 (0.030, 1.775)	0.159			
White blood cell count > 9.5 ×10^9^/L	0.751	2.119 (0.864, 5.197)	0.101			
Neutrophils > 6.3 ×10^9^/L	1.099	3.000 (1.328, 6.780)	**0.008**	1.185	3.271 (1.268, 8.440)	**0.014**
Low HDL-c (<1.0 mmol/L for male; <1.2 mmol/L for female)	0.011	1.011 (0.465, 2.201)	0.977			
QTc>440 ms	0.44	1.552 (0.681, 3.538)	0.295			

*APS, antiphospholipid syndrome; AMI, acute myocardial infarction; HDL, high-density lipoprotein cholesterol. The bold values means significantly different (p <0.05)*.

### Overall Survival and Recurrence of Thrombosis

**Figure 2:**
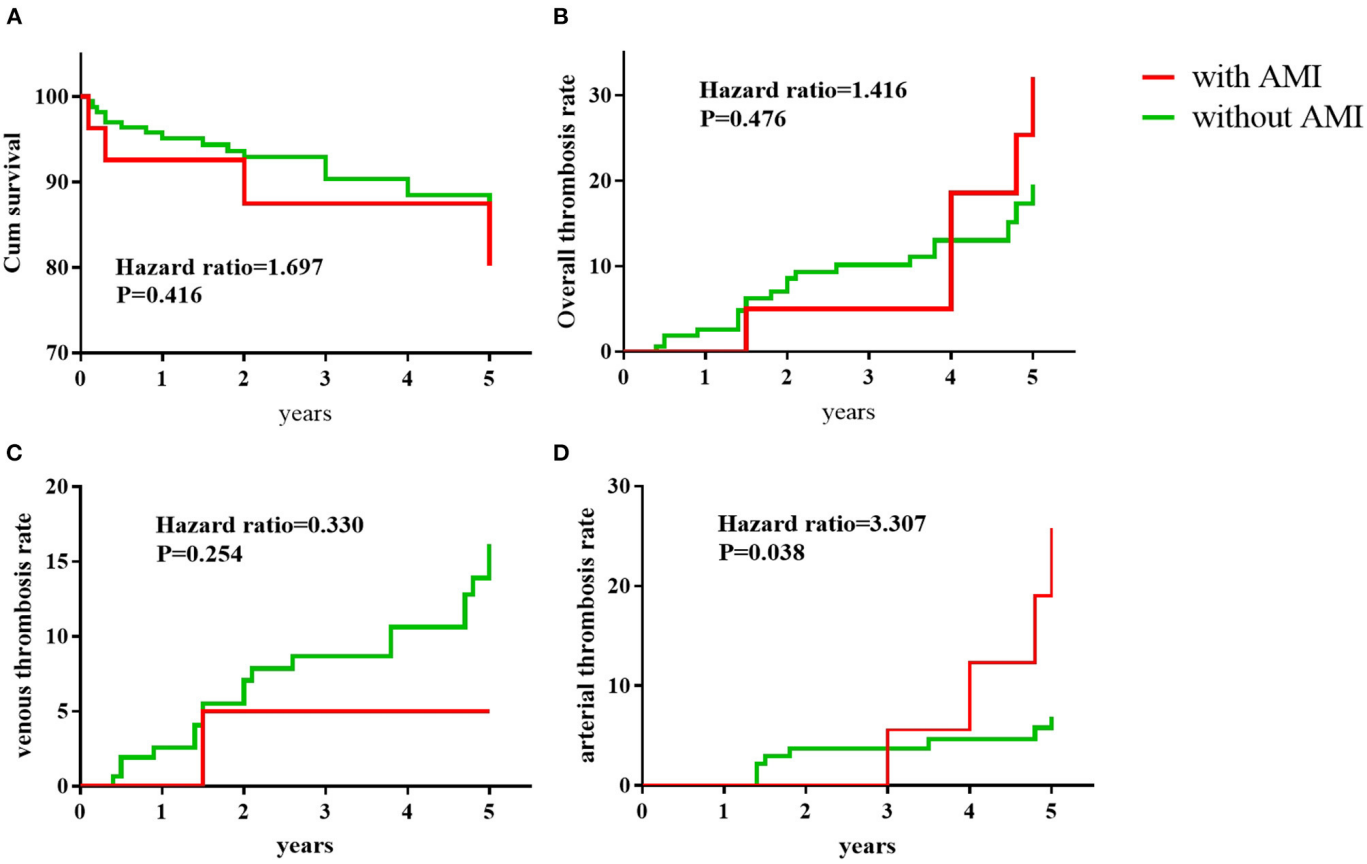
Clinical outcomes during the 5-year follow-up. Kaplan–Meier analysis of the portion of APS patients remaining survival **(A)**, with overall recurrent thrombosis **(B)**, recurrent venous thrombosis **(C)**, and recurrent arterial thrombosis **(D)**. *P-*values were calculated with the use of the stratified log-rank test.

The median follow-up duration was 4.8 years in the AMI group and 4.5 years in the non-AMI group. At the last follow-up visit, 4/27 patients in the AMI group and 17/167 in the non-AMI group died, and overall survival rates between the two groups were not significantly different (Figure 2A). Detailed causes of death in these 21 patients with APS were shown in Table 5. In the AMI group, two patients died of pulmonary embolism and the other two patients died of severe infection. In the non-AMI group, the patients died of multiple causes, including pulmonary embolism (*n* = 5), severe infection (*n* = 2), malignant tumors (*n* = 2), severe thrombocytopenia (*n* = 1), disseminated intravascular coagulation (*n* = 1), and macrophage activation syndrome (*n* = 1).

**Table 5:** Cause of death in 21 APS patients.

**Cause**	**With AMI (*n* = 4)**	**Without AMI (*n =* 17)**
Pulmonary embolism	2	5
Sever infection	2	7
Malignancy		2
Sever hemorrhage		1
Disseminated Intravascular Coagulation		1
Macrophage Activation Syndrome		1

*APS, antiphospholipid syndrome; AMI, acute myocardial infarction*.

As shown in Table 6, APS patients with AMI received more antiplatelet treatments [17 (63.0%) vs. 44 (26.3%), *p* < 0.001] and fewer immunosuppressants [10 (37.0%) vs. 123 (73.6%), *p* < 0.001] compared to APS patients without AMI. The application of anticoagulants, hydroxychloroquine, and corticosteroids was not significantly different between the two groups. The mean INR of APS patients who took the vitamin K antagonist was not significantly different between the AMI group and non-AMI group (2.19 ± 0.89 vs. 2.24 ± 0.68, *P* = 0.766). During the 5-year follow-up, the recurrence rate of overall thrombosis was 18.6% (5/27) in the AMI group and 13.7% (23/167) in the non-AMI group; recurrence of venous thrombosis was 3.7% (1/27) in the AMI group and 11.3% (19/167) in the non-AMI group, and the recurrence of arterial thrombosis was 14.8% (4/27) in the AMI group and 4.8% (8/167) in the non-AMI group, respectively. Kaplan–Meier analysis showed that there was no significant difference between the AMI group and the non-AMI group in recurrent rates of overall thrombosis (Figure 2B) or venous thrombosis (Figure 2C). However, the recurrence rates of arterial thrombosis differed significantly between the two groups (Figure 2D).

**Table 6:** Medication of 194 APS patients during follow-up.

**Medication**	**Overall** **(*n* = 194)**	**with AMI** **(*n* = 27)**	**without AMI (*n* = 167)**	**p**
Sustained anticoagulants*, n* (%)	133 (68.5)	17 (63.0)	116 (69.5)	0.627
VKA, *n* (%)	99 (74.4)	13 (76.4)	86 (74.1)	0.448
DOACs, *n* (%)	26 (19.5)	3 (17.6)	23 (19.8)	
LMWH, *n* (%)	8 (6.0)	1 (5.8)	7 (6.0)	
Antiplatelet, *n* (%)	61 (31.4)	17 (63.0)	44 (26.3)	**<0.001**
Hydroxychloroquine, *n* (%)	179 (92.3)	19 (70.4)	160 (95.8)	0.159
Corticosteroids, *n* (%)	185 (94.8)	21 (77.8)	164 (98.2)	0.353
Other immunosuppressants, *n* (%)	133 (68.6)	10 (37.0)	123 (73.6)	**<0.001**

*APS, antiphospholipid syndrome; AMI, acute myocardial infarction; VKA, Vitamin K antagonist; DOACs, direct oral anticoagulants; LMWH, low-molecular-weight heparin. The bold values means significantly different (p <0.05)*.

## Discussion

Our study showed that patients with APS had a high risk of AMI due to thromboembolism and accelerated atherosclerosis. To our knowledge, this is the first cohort study to characterize and explore the risk factors for AMI in patients with APS.

Previously, two register-based studies reported that 1.9–2.8% of patients with APS may develop myocardial infarction (3, 13). However, in our cohort, 12.1% of APS patients developed AMI. The explanation for the higher incidence in our cohort could be the different study designs; these two studies only included AMI events occurring after diagnosis of APS, whereas we included all AMI events which occurred both before the diagnosis of APS and during the follow-up. Another explanation could be the difference in ethnic backgrounds. In our study cohort, all patients were Han Chinese but all patients were Caucasians in two previous studies. In addition, Sacré et al. discovered that the prevalence of myocardial scarring detected by cardiac MRI was 11%, indicating that more than one-tenth of the patients with APS underwent ischemic cardiac events. Andreoli et al. estimated that the overall aPL frequencies in MI were 11% (14). Taken together, we confirmed an increased incidence of AMI in patients with APS.

Early studies reported that 90% of patients with AMI have obvious coronary artery obstruction (12). Recently, MINOCA has been increasingly recognized owing to the common use of high-sensitivity troponins and coronary angiography. Although the American Heart Association emphasized that it is necessary to exclude causes of spontaneous thromboembolism, such as APS (15), the exact incidence of MINOCA in APS remains unclear. In our cohort, MINOCA was seen in one-third of AMI patients, but a systematic study showed that normal coronaries were seen in 75% of APS patients with AMI by cardiac catheterization (16). Such a high incidence might partially be because the case series included in the review mainly focused on MINOCA. In addition, Tornvall et al. discovered that the incidence of MINOCA was similar in systemic lupus erythematosus (SLE) and non-SLE controls (4). Moreover, we found that atherosclerosis, with a higher percentage in the AMI group, could serve as a unique risk factor for AMI in patients with APS. Taken together, our present study suggests that despite MINOCA being common, the majority of AMI cases in APS are attributed to atherosclerosis.

Apart from atherosclerosis, we did not observe any other traditional CVD risk factors (smoking, diabetes mellitus, hypertension, dyslipidemia, chronic kidney disease, and hyperuricemia) that contributed to AMI in our APS cohort. Such phenomena are also found in SLE. Increased risk of ischemic cardiac events in SLE cannot be solely explained by traditional Framingham cardiovascular risk factors (7, 17). These observations prompt us to focus on disease-related risk factors instead of traditional CVD risk factors. It is known that antiphospholipid antibodies are associated with hypercoagulability and myocardial infarction (8, 14, 18, 19). We discovered that patients with AMI had multiple organ thrombosis and recurrent thrombosis, and recurrent thrombosis was a risk factor for AMI, indicating that AMI in APS might be associated with a more severe thrombophilic condition (1, 20). Despite a higher percentage of antiplatelet treatment, the incidence of subsequent arterial thrombosis was significantly higher in the AMI group. Therefore, our study highlights the importance of sustained anticoagulants for the treatment of AMI in APS in addition to controlling cardiovascular risk factors and antiplatelet therapy (6, 21).

Interestingly, by multiple logistic analyses, we revealed that neutrophil elevation was the only laboratory index associated with AMI. Active immunological responses and inflammation have been proposed as important triggers for endothelial dysfunction, leading to accelerated atherosclerosis and cardiovascular diseases in autoimmune diseases (1, 7, 17). In APS patients, neutrophils display an activated phenotype with increased aggregation and mitochondrial dysfunction by increasing mitochondrial reactive oxygen species (ROS) production and enhanced spontaneous neutrophil extracellular trap release, which leads to hyper-inflammation and over-production of antiphospholipid antibodies. It indicates that neutrophils play an important role in the pathogenesis of APS (22, 23). A recent study also found that incased neutrophil count could serve as a biomarker for APS in SLE (24). In addition, compelling evidence indicates a pivotal pathogenic role for neutrophils in acute coronary syndrome (25–27). These findings suggest that elevated neutrophil levels could serve as biomarkers for predicting AMI in APS. However, Pabinger et al. recently reported different phenotypes between neutrophil subpopulations [high and low-density neutrophils (HDNs/LDNs)] in APS (28), suggesting that further work might focus on different subtypes of neutrophils. In addition, decreased neutrophil levels appear to have a better prognosis in myocardial infarction (29, 30), suggesting that neutrophils could be a target for the prevention of AMI in APS. Patients with AMI would be expected to have higher C reactive protein (CRP) levels. However, CRP levels are more elevated in the group without AMI, without statistical significance. This phenomenon might be due to several reasons. Firstly, some patients were not in the acute phase of AMI when they were enrolled in our cohort (31). In addition, some previous studies assumed that hsCRP is more accurate in predicting CV events than CRP. Unfortunately, the hsCRP is not a routine test in our Department.

It is well-accepted that the hallmark of APS is the presence of thrombotic events (8). However, the majority of previous studies only focus on the characteristics and risk factors for overall thrombosis events (32–34), rarely subdividing thrombotic APS into arterial and venous. It is recognized that venous and arterial thrombotic disorders are mechanistically and pathophysiologically distinct entities (35). A systematic review revealed the types of antiphospholipid antibodies that were different in arterial thrombosis and venous thrombosis, thus arterial thrombosis showed higher levels of anticardiolipin antibodies and the lupus anticoagulant (36). Recently, Savino et al. found that levels of IgG anti-high-density lipoproteins antibodies were only associated with arterial thrombosis (37). Moreover, Freire et al. discovered that thrombotic microangiopathy in APS was characterized by an increased frequency of arterial events and stroke and less deep venous thrombosis (38). Similarly, we discovered that the pre-venous thrombosis event was negatively associated with AMI, and AMI predicted a higher incidence of subsequent arterial thrombosis. In addition, prevention strategies have shown some differences between arterial and venous thrombosis (39). These findings suggest that distinct pathophysiological mechanisms may exist and contribute to the development of venous or arterial thrombotic APS, and we may need to pay more attention to arterial thrombotic APS in cases of CVD.

### Study Limitation

This study has several limitations. First, due to the relatively small number of patients with AMI, we did not compare the clinical and laboratory differences among UA, STEMI, and NSTEMI, and the effectiveness of different therapies in the acute phase of AMI, despite the lower percentage of invasive treatment. Second, because of the limitations of this retrospective study, we did not discuss the monitoring function of neutrophils or the therapeutic role of neutrophil-targeting therapy in AMI. Therefore, prospective cohort or multicenter studies are needed in the future.

## Conclusion

In the present study, we investigated clinical and laboratory features of AMI in patients with APS and discovered that atherosclerosis, multiple organ thrombosis, and elevated neutrophil levels may be risk factors for AMI. Arterial thrombotic disorders might be different from venous thrombosis in APS, as venous thrombosis is negatively associated with AMI. AMI only predicts the subsequent recurrence of arterial thrombosis. Future prospective or multicenter studies are desired to validate our findings.

## Data Availability Statement

The datasets presented in this article are not readily available because it contains sensitive information that could compromise patient anonymity and contain sensitive medical information. Requests to access the datasets should be directed to fiona_leechun@163.com.

## Ethics Statement

The studies involving human participants were reviewed and approved by Ethics Committee of Peking University People's Hospital. The patients/participants provided their written informed consent to participate in this study.

## Author Contributions

YZG and YWZ: data interpretation and analysis, writing of the original draft, review, and editing. GML: clinical data collection. JPG: editing. HY and YSZ: follow-up of participants. CH and LW: interpretation and collection of cardiac parameter data. CL: conceptualization, methodology, investigation, resources, data curation, supervision, manuscript editing, and funding acquisition. All authors have read and approved the final manuscript.

## Funding

This work was supported by the National Natural Science Foundation of China (Nos. 81801615, 81871289, and 82071814), University of Michigan Medical School (UMMS) and Peking University Health Science Center (PUHSC) Joint Institute (JI) Projects (No. BMU2020JI003), Peking University Medicine Fund of Fostering Young Scholars' Scientific and Technological Innovation and Fundamental Research Funds for the Central Universities (BMU2022PY004), and Peking University People's Hospital Research and Development Funds (RDY 2019-04).

## Conflict of Interest

The authors declare that the research was conducted in the absence of any commercial or financial relationships that could be construed as a potential conflict of interest.

## Publisher's Note

All claims expressed in this article are solely those of the authors and do not necessarily represent those of their affiliated organizations, or those of the publisher, the editors and the reviewers. Any product that may be evaluated in this article, or claim that may be made by its manufacturer, is not guaranteed or endorsed by the publisher.
