# Supplementary material for: Risk Factors and Outcomes of Acute Myocardial Infarction in a Cohort of Antiphospholipid Syndrome
Source: Front Cardiovasc Med. 2022 Jul 5;9:871011. doi: 10.3389/fcvm.2022.871011 (PMC9294316; doi:10.3389/fcvm.2022.871011)
Supplement: Supplementary file 1 [file Table_1.DOCX]

**Table S1. Detailed profiles of accompanied autoimmune diseases**

|  | With AMI (n=16) | | Without AMI (n=128) | |  |
| --- | --- | --- | --- | --- | --- |
| Systemic lupus erythematosus (SLE) | 9 | 93 | | | |
| Sjogren syndrome (SS) | 3 | 6 | |  |  |
| SLE + SS | 1 | 10 | |  |  |
| Undifferentiated Connective Tissue Disease | 1 | 2 | |  |  |
| Rheumatoid arthritis | 0 | 7 | |  |  |
| Systemic sclerosis | 1 | 2 | |  |  |
| Behcet disease | 0 | 6 | |  |  |
| Takayasu arteritis | 1 | 1 | |  |  |
| ANCA associated Vasculitis | 0 | 1 | |  |  |
